# Neonatal tooth with Riga-Fide disease affecting breastfeeding: a case report

**DOI:** 10.1186/s13006-018-0176-7

**Published:** 2018-07-27

**Authors:** Nurjasmine Aida Jamani, Yunita Dewi Ardini, Nor Asilah Harun

**Affiliations:** 1 Department of Family Medicine, Kulliyyah of Medicine, International Islamic University Malaysia, Kuantan, Pahang Malaysia; 2 Department of Paediatric Dentistry and Dental Public Health, International Islamic University Malaysia, Kuantan, Pahang Malaysia

**Keywords:** Neonatal tooth, Breastfeeding difficulties, Sublingual ulceration, Riga-Fede disease

## Abstract

**Background:**

Neonatal teeth erupt during the neonatal period and natal teeth are the presence of teeth since birth. While rare, natal teeth and neonatal teeth can have a significant impact on breastfeeding. Neonatal teeth are less common, and although its exact etiology is still unknown, it can cause difficulties in breastfeeding to the mother and may eventually lead to discontinuation of breastfeeding. Other associated possible complications include tooth aspiration and sublingual ulceration. This paper was aimed to discuss the clinical features, complications, and management of neonatal tooth, in addition to its impact on breastfeeding and role in sublingual ulcer formation.

**Case presentation:**

We present a baby girl who had a neonatal tooth with sublingual ulceration (Riga-Fede disease), which resulted in a difficulty to breastfeed for the baby and nipple pain to the mother. Following the extraction of the baby’s tooth, she immediately continued breastfeeding, and her tongue ulcer healed well.

**Conclusion:**

Extraction of the neonatal tooth promoted rapid healing of oral ulcers and the reestablishment of breastfeeding.

## Background

Natal teeth are defined as the presence of teeth at birth, which usually erupt during intrauterine life. Meanwhile, neonatal teeth refer to the eruption of teeth during the first 4 weeks of life [1, 2]. Its incidence has been reported to be around 1:800 to 1:6000 births [2, 3]. The etiology of this condition is still inconclusive [1]. According to several studies, this condition was more common in females but the majority of researches have reported an absence of gender predilection [3].

Milk teeth or primary teeth normally erupt around 6 months of age. Neonatal teeth erupt prior to this period (especially during intrauterine life or within the first 4 weeks of life), which can pose a problem to both mother and baby especially in terms of breastfeeding. Neonatal teeth can give rise to suckling problems, choking risks, and soft tissue injuries in the baby, as well as nipple pain in the mother [3, 4].

One of the well-known complications of neonatal teeth is sublingual ulceration, or Riga-Fede disease, which are the result of repetitive trauma to the area. Antonio Riga, an Italian physician, first described the lesion in 1881; subsequently, F. Fede published the histological findings of the ulcer in 1890 [4]. Ever since, this condition has been assigned various eponyms such as Riga’s disease, Riga-Fede disease or syndrome, sublingual ulcer, sublingual granuloma, and traumatic sublingual ulceration.

There are myths concerning babies who are born with teeth. In certain parts of the world like Malaysia, neonatal teeth are considered as a source of luck. However, in other places, these are considered to be a bad omen [3, 4]. The management of neonatal teeth, varies from center to center and ranges from conservative to surgical interventions, which includes the tooth extraction.

This paper concerned a 1 month old neonate who presented with feeding difficulties, and was subsequently diagnosed with a neonatal tooth complicated by Riga-Fede disease. The clinical presentation, progress, and management of this patient are described as follows.

## Case presentation

A one month old baby girl was brought by her mother to the lactation clinic for further evaluation due to the difficulty of breastfeeding. The mother complained of pain in the nipple which was present throughout the entire breastfeeding session and usually recurred with each episode of breastfeeding. Two weeks after delivery, the mother noticed a tooth on the baby’s lower left gum. A week later, she noticed an ulcer under her baby’s tongue, which occasionally bled.

In light of the continuous pain during breastfeeding, the mother became reluctant to continue and infant formula milk was given as a substitute. Self-examination by the mother did not reveal any nipple crack or breast injury. Her antenatal history was uneventful. There was strong family history of natal teeth, and this baby’s siblings had similar problems.

On examination, the mother’s breast appeared to be normal and consistent with a lactating breast. There was no inflammation or engorgement. Some mild eczema was noted around the nipple, but there was no infection. Cervical and axillary lymph nodes were not palpable.

**Fig. 1:**
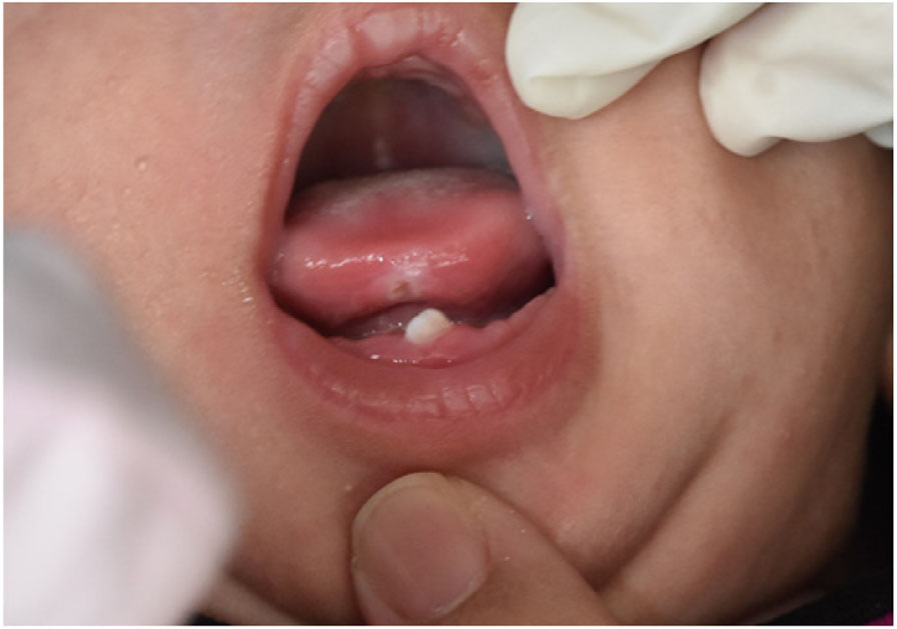
Neonatal tooth erupting from the lower gum at one month

**Fig. 2:**
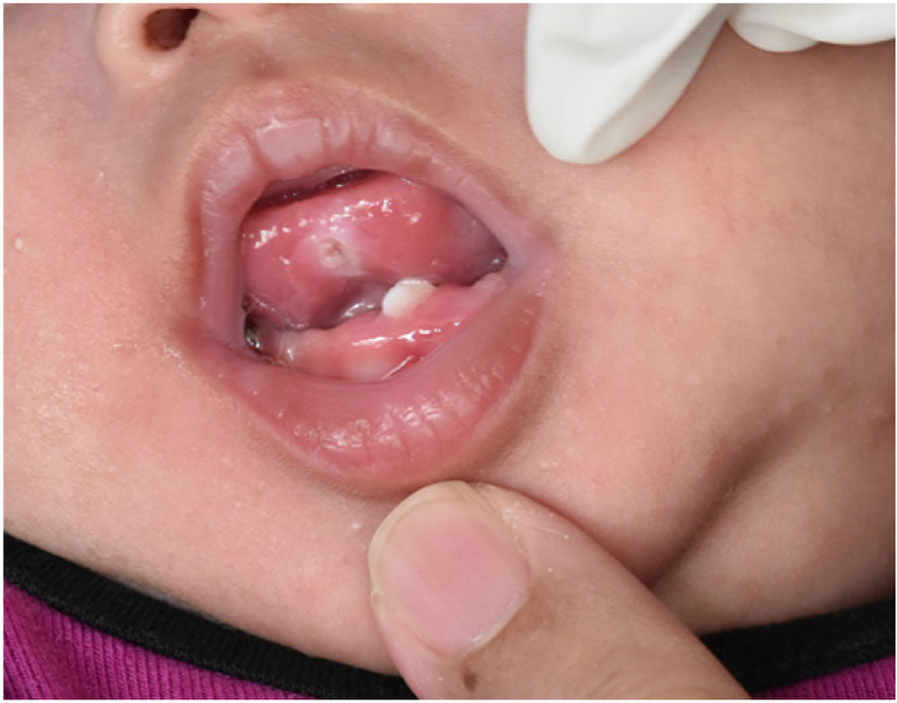
Ulceration at the ventral aspect of the tongue (Riga Fide disease) at one month

Examination of the oral cavity of the baby revealed a neonatal tooth over the left anterior region of the mandibular ridge. The tooth measured 2 mm × 1 mm in size, was whitish opaque, and had Grade II mobility (Fig. 1). There was also a whitish ulcer over the ventral aspect of the tongue measuring 1 mm × 1 mm (Fig. 2). The parents declined radiological investigations and tongue biopsy for the baby. Hence, the clinical diagnosis was neonatal tooth associated with Riga-Fede disease causing difficulty in breastfeeding.

The baby was then referred to the pediatric dental surgery department. After a thorough discussion with the parents, the neonatal tooth was extracted under local anesthesia. The tooth had a crown but lacked a root. Following extraction, the baby did not have any complication such as bleeding and infection. The wound healed well within 2 days and she successfully resumed taking breast feeds.

**Fig. 3:**
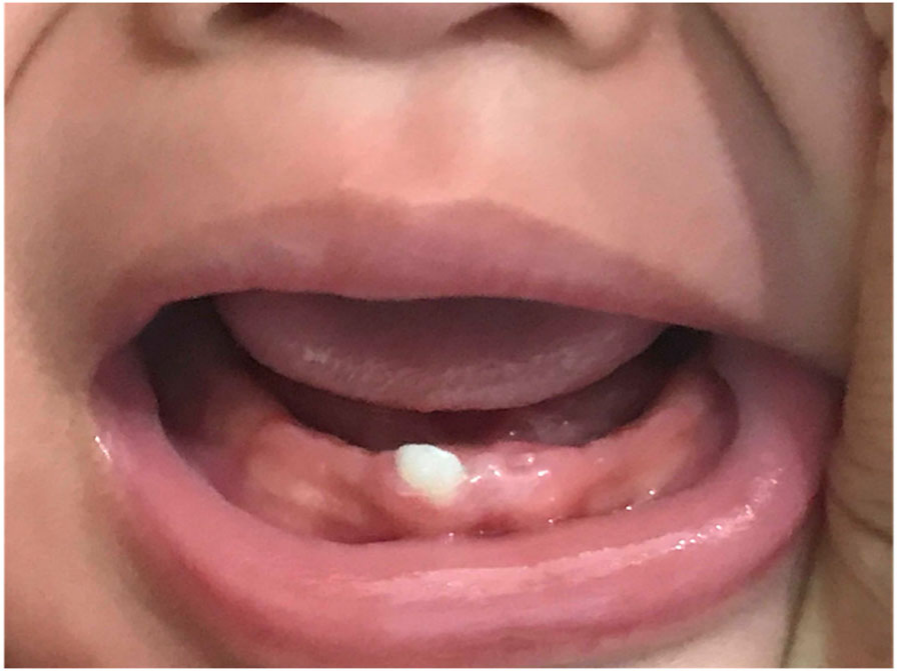
Appearance of oral cavity 10 months after extraction

Currently, at 10 months post-extraction, the child is growing well and still breastfeeding. Examination of the oral cavity revealed that only one central lower incisor tooth was present (Fig. 3).

## Discussion

Normally, primary teeth erupt around 6 months of age, with the incisors being the first to do so [5]. However, for natal/neonatal teeth, the mandibular central incisors are the most common, followed by maxillary incisors, mandibular cuspids or molars, and maxillary cuspids or molars [3, 6]. Previous studies have found that most natal and neonatal tooth are primary teeth rather than supernumerary teeth [6]. In the case of our patient, the tooth was a mandibular incisor.

Although the exact cause of this condition is still unknown, many etiological theories have been postulated. These included maternal exposure to environmental toxins, febrile states, infections, malnutrition, hormonal stimulation, trauma, osteoblastic activity in the germ zone, and congenital syndromes [4, 5]. Some authors have suggested that genetics could be a causative factor in light of several reports on the possible role of an autosomal dominant gene in the pathogenesis. For example, Kates et al. (1984) found that 7 of 38 cases of natal/neonatal teeth had a positive family history of the same [7]. As with our case, the siblings of the baby also had this condition.

Neonatal teeth are usually small and conical, but can assume the sizes and shapes of normal teeth [4]. They may be yellowish or brownish and are usually immature, with enamel hypomineralisation and small roots [4, 5]. According to the classification by Spoug and Feasby (1966), which evaluated the maturity and appearances of neonatal and natal teeth, these types of dentition have a poor outcome [8].

The differential diagnosis of neonatal tooth includes dental lamina cysts and hamartoma. If a neonatal tooth is located at the posterior part of the mandible, lymphangioma needs to be excluded [9, 10].

Possible complications that are associated with natal or neonatal teeth are tooth aspiration, sublingual ulceration, and a difficulty with breastfeeding. Ulceration to the mother’s nipple is a cause for concern as well. Moura et al. (2013) reported that two of 23 cases of natal teeth had breastfeeding difficulties [9].

However, Zhu and King (1995) did not find an association between the presence of neonatal tooth and injury to the mother’s nipple by being bitten. This was in view of the fact that the tongue is interposed between the teeth and the nipple during breastfeeding [10]. Although the nipple can reach the posterior part of the baby’s mouth, the baby’s tongue covers the lower gum while the lips and gums touch the maternal areola. Therefore, even with presence of teeth, the baby is unable to bite during actively breastfeeding [11].

Sublingual ulceration, or Riga-Fede disease, is a lesion of the mucosa of the tongue which arises following repetitive trauma by the tooth during tongue movements. The ulcer most commonly presents at the ventral aspect of the tongue, although other parts can be affected as well [12, 13]. Persistent trauma may create a sufficiently severe ulcer that interferes with effective suckling of the mother’s milk. Failure to diagnose this lesion can lead to tongue deformities, dehydration, and inadequate nutrition intake, all of which eventually result in poor growth as well as development in the child.

History and physical examination are sufficient to make a diagnosis of Riga-Fede disease since the clinical features are so typical that there is rarely a need for additional histopathological investigations [13]. As with this case, the diagnosis was made with reference to the typical features of the same. An accurate diagnosis of neonatal tooth is crucial, and clinical and radiographic imaging can be used to distinguish between normal and supernumerary dentition [5, 14]. One advantage of radiography is that it can verify the presence or absence of tooth germ in the area of the primary teeth [14].

The management of this condition can be a challenge since there is a debate between conservative treatment and tooth extraction. Tooth extraction is considered in the following cases: (i) mobile teeth, (ii) injury to the tongue and adjacent soft tissues, (iii) interference with breastfeeding, as well as (iv) supernumerary teeth [9, 14]. Nevertheless, a few studies have recommended against tooth extraction. Conservative treatment modalities, which involve measures like grinding the sharp edges or placing a composite resin, have been practiced [4, 12]. However, neonatal teeth should definitely be extracted if conservative treatment fails or if the tooth is loose, because the latter can lead to aspiration [13].

The tooth of our patient had a solid crown which was poorly fixed to the alveolus by the gingival tissue. Apart from having no root, it also exhibited Grade II mobility [15]. In the presence of these features, the baby was at risk of tooth aspiration. Additionally, the presence of an ulcer on the ventral aspect of the tongue, as well as difficulty in breastfeeding, resulted in the decision to extract the neonatal tooth. The benefit of this measure was that the baby was able to receive breastfeeds for longer periods without interruption. Even though radiographic imaging was likely to be useful in this baby, the management would remain the same.

## Conclusion

While neonatal teeth are rare, their occurrence can result in sublingual ulceration (Riga-Fede disease) and interfere with breastfeeding. This condition should be assessed properly and managed independently in order to come up with the best treatment option, apart from minimising the likelihood of a poor weight gain in the infant. Extraction is a viable option if the tooth is mobile or when associated complications are present. This measure also allows immediate continuation of breastfeeding, prevents growth and nutritional deficiency, as well as enables effective healing of oral and tongue ulcers.
